# Epizootic Hemorrhagic Disease Virus Serotype 6 Infection in Cattle, Japan, 2015

**DOI:** 10.3201/eid2405.171859

**Published:** 2018-05

**Authors:** Yuka Kamomae, Masahiro Kamomae, Yasuyuki Ohta, Mikoto Nabe, Yuichi Kagawa, Yuji Ogura, Tomoko Kato, Shogo Tanaka, Tohru Yanase, Hiroaki Shirafuji

**Affiliations:** Awaji Livestock Hygiene Service Center, Minamiawaji, Japan (Y. Kamomae, Y. Ohta, Y. Kagawa);; Himeji Livestock Hygiene Service Center, Himeji, Japan (M. Kamomae, M. Nabe, Y. Ogura);; National Agriculture and Food Research Organization, Kagoshima, Japan (T. Kato, S. Tanaka, T. Yanase, H. Shirafuji)

**Keywords:** arboviruses, cattle, *Orbivirus*, *Reoviridae*, epizootic hemorrhagic disease virus, viruses, serotype 6, livestock, Japan, epizootic disease, vector-borne infections

## Abstract

During October–December 2015, an epizootic hemorrhagic disease outbreak occurred in cattle in Japan. Forty-six animals displayed fever, anorexia, cessation of rumination, salivation, and dysphagia. Virologic, serologic, and pathologic investigations revealed the causative agent was epizootic hemorrhagic disease virus serotype 6. Further virus characterization is needed to determine virus pathogenicity.

Epizootic hemorrhagic disease virus (EHDV; genus *Orbivirus*, family *Reoviridae*) is an arthropodborne virus that is transmitted among ruminant hosts by the bite of *Culicoides* midges (*1**–**3*). EHDV infection of wild and domestic ruminants has been reported in the Americas, Africa, Asia, Australia, the Middle East, and some islands of the Indian Ocean (*1*) and affects primarily white-tailed deer (*Odocoileus virginianus*) and cattle (*1**–**3*). EHDV infection in cattle does not usually result in clinical disease, but clinical cases of epizootic hemorrhagic disease in cattle have been reported in several countries in Asia, Africa, and the Middle East (*1**–**5*).

EHDV serotype 2 (EHDV-2) strain Ibaraki virus was first identified in affected cattle in Japan in 1959. Ibaraki virus has caused disease repetitively in cattle, with signs and symptoms including fever, anorexia, nasal and ocular discharge, congestion of conjunctival and nasal mucous membranes, swollen eyelids and tongue, and dysphagia (*4**,**6*). A large-scale epidemic of atypical Ibaraki disease also occurred in Japan in 1997; the epidemic caused mainly abortion and stillbirth in cattle, and the causative agent was found to be EHDV-7 (*7**,**8*). Several strains of EHDV-1, EHDV-7, and EHDV-10 were also observed in Japan but did not appear to be associated with clinical disease in cattle (*8*).

In October 2015, an outbreak of febrile illness occurred in cattle in Hyogo Prefecture, Japan, and lasted 3 months. We summarize the features of this outbreak, which affected 46 cattle at 38 farms.

## The Study

Of the 46 affected cattle, 40 were beef cattle (Japanese Black) and 6 were dairy cattle (Holstein heifers). The average age of all affected animals was 114.7 (range 11–187) months. The clinical signs observed in beef cattle were fever, anorexia, cessation of rumination, swollen eyelids, salivation, paralysis of the tongue, difficulty swallowing, nasal and ocular discharge, and abortion (Table 1). Seven beef cattle died, and 2 were euthanized because they did not recover despite treatment of symptoms. The dairy cattle showed fever, anorexia, coughing, conjunctivitis, cessation of rumination, salivation, difficulty swallowing, and reduced milk production.

**Table 1:** Clinical manifestations of affected cattle during epizootic hemorrhagic disease outbreak, Japan, 2015

Clinical sign	No. cattle
Anorexia	38
Fever	28
Cessation of rumination	22
Salivation	20
Difficulty swallowing	19
Swollen eyelids	8
Coughing	3
Conjunctivitis	3
Reduced milk production	3
Abortion	2
Ocular discharge	1
Death	9*

*Includes 2 euthanized cattle.

Early in the outbreak, Ibaraki disease was suspected, so blood samples were collected. We washed the blood cells and used them for virus isolation and reverse transcription PCR (RT-PCR) for group-specific and serotype-specific EHDV detection (*9**,**10*). Paired serum samples were also collected from 20 of the 46 affected animals at early onset of the outbreak and after the outbreak at 1- or 2-month intervals. We used the paired serum samples for neutralization tests and conducted necropsies on 2 euthanized animals.

All 46 of the affected animals tested positive for EHDV by group-specific RT-PCR (*9*) and EHDV-6 by serotype-specific RT-PCR (*10*), although we did not isolate infectious virus. The neutralizing antibody titers of serum samples acquired after the outbreak (n = 20) were 1:>32 for EHDV-6 strain AUS1981/07 CSIRO 753 (Table 2). The neutralization test also showed increases (>4-fold) in EHDV-6 antibody titers for paired serum samples of 5 cattle (nos. 6, 7, 10, 11, and 13), although such increases were not observed in the other 15 cattle (Table 2).

**Table 2:** Neutralizing antibody titers against epizootic hemorrhagic disease virus serotype 6 in paired serum samples collected from affected cattle, Japan, 2015

No.	Early onset	After outbreak
1	1:32	1:64
2	1:64	1:32
3	1:32	1:32
4	1:32	1:64
5	1:64	1:32
6	1:8	1:64
7	1:16	1:64
8	1:64	1:64
9	1:32	1:64
10	1:2	1:64
11	<1:2	1:64
12	1:64	1:128
13	1:32	1:256
14	1:64	1:128
15	1:32	1:32
16	1:32	1:32
17	1:16	1:32
18	1:128	1:32
19	1:16	1:32
20	1:32	1:32

**Figure 1:**
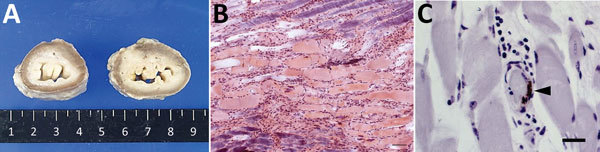
Lesions and epizootic hemorrhagic disease virus serotype 6 (EHDV-6) antigen in esophagus of necropsied cow, Japan, 2015. A) Dilation of lumen. Cross-section of formalin-fixed esophagus of affected cow (right) and control (left). B) Hyaline degeneration of striated muscle accompanied by cell infiltration. Phosphotungstic acid–hematoxylin stain. Scale bar indicates 50 μm. C) EHDV-6 antigen (arrowhead) in vascular endothelium in esophageal muscularis externa. Immunohistochemical stain. Scale bar indicates 20 μm.

At necropsy, we observed edema of the esophagus, pharynx, and tongue in an affected cow with dysphagia. The lumen of the esophagus was also dilated (Figure 1, panel A). The histopathologic lesions included hyaline degeneration and necrosis of striated muscle accompanied by cell infiltration in the esophagus and tongue (Figure 1, panel B), thrombosis of small vessels, a proliferation of connective tissue in the esophagus, and necrosis of striated muscle in the pharynx. We conducted immunohistochemical assays with necropsy tissue samples from this cow using rabbit antiserum against EHDV-6 AUS1981/07 CSIRO 753 and detected antigen in the vascular endothelium of the esophageal muscle layer (Figure 1, panel C).

We further characterized the causative agent by performing sequence analysis of genome segment 2, which correlates with serotype, and segment 3, which correlates with geographic genetic type, of outbreak isolate HG-1/E/15. We designed RT-PCR primers to amplify segments 2 and 3 from the full-length cDNA on the basis of sequence data of EHDV strains from Japan and Australia available in GenBank. We performed RT-PCR using RNA obtained from an affected cow (no. 9; Table 2) as template; we then purified the RT-PCR products and subjected them to direct sequencing. We used the sequence data to analyze the phylogenetic relationships between EHDV HG-1/E/15 and other EHDV isolates.

**Figure 2:**
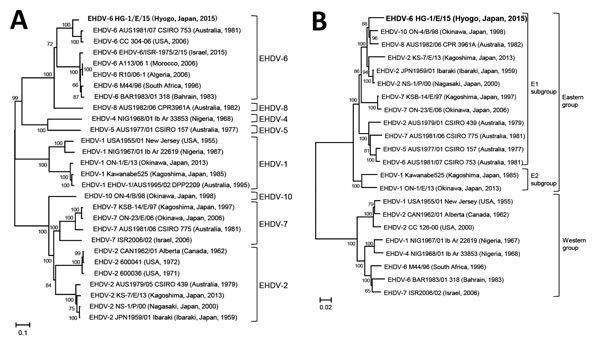
Phylogenetic profile of HG-1/E/15 from EHDV outbreak in cattle, Japan, 2015, compared with reference viruses. A) Phylogenetic profile on the basis of coding region segment 2. EHDV HG-1/E/15 (bold; 2,919 bp) clustered with EHDV-6 strains. B) Phylogenetic profile on the basis of segment 3. EHDV-6 HG-1/E/15 (bold; 2,700 bp) clustered with E1 subgroup of the Eastern group. Virus strain names and location and year of isolation are provided. Scale bars indicate nucleotide substitutions per site. EHDV, epizootic hemorrhagic disease virus.

We aligned the sequences using ClustalW (*11*) and constructed phylogenetic trees with MEGA5 using the neighbor-joining method (*12*); the reliability of the branching orders was evaluated by the bootstrap test (1,000 replicates). Sequence identities between EHDV HG-1/E/15 and the other EHDV isolates were calculated with GENETYX version 10 (GENETYX Corporation, Tokyo, Japan). The results of the phylogenetic analysis of segment 2 (DDBJ accession no. LC320035) revealed that HG-1/E/15 clustered with EHDV-6 (Figure 2, panel A) and showed highest identity to EHDV-6 AUS1981/07 CSIRO 753 (89.68% nucleotide and 93.51% amino acid identities). In contrast, the phylogenetic analysis of segment 3 (DDBJ accession no. LC320036) showed that EHDV-6 HG-1/E/15 sorted into the E1 subgroup of the Eastern group (Figure 2, panel B) (*8*) and had highest identity to the Ibaraki isolate EHDV-2 JPN1959/01 (95.96% nucleotide and 99.77% amino acid identities).

## Conclusions

We determined this febrile illness affecting 46 cattle in Japan in 2015 was an epizootic hemorrhagic disease outbreak caused by EHDV-6 infection on the basis of clinical findings, RT-PCR results, neutralization tests, and sequence analyses. Clinical EHDV-6 cases in cattle have been reported in Turkey, Bahrain, Israel, Morocco, Tunisia, Algeria, and Réunion Island (*3**,**5**,**13**,**14*), but the EHDV-6 detected in this outbreak (HG-1/E/15) clustered separately from the EHDV-6 isolates from Africa and the Middle East in the phylogenetic tree analysis of segment 2 (Figure 2, panel A). Because HG-1/E/15 clustered with EHDV isolates from Japan and Australia in the E1 subgroup in the phylogenetic analysis of segment 3 (Figure 2, panel B) (*8*), HG-1/E/15 appears to be derived from EHDVs circulating in the Asia-Pacific region.

The neutralization tests showed significant increases of antibody titers in 5 cattle, suggesting recent EHDV-6 infection. At the same time, no significant increase of antibody titers was observed with the other 15 cattle. These results suggest that the onset of the epizootic hemorrhagic disease outbreak might have occurred >3–5 weeks after the EHDV-6 infection in the 15 seronegative cattle, considering that neutralizing antibody titer increases have been observed to occur in cattle 11–14 to 21–37 days after experimental EHDV-6 infection (*15*).

The clinical and pathologic findings of this outbreak are similar to those observed for Ibaraki disease in cattle. Thus, EHDV-6 HG-1/E/15 possibly has pathogenic characteristics similar to those of Ibaraki strain EHDV-2 JPN1959/01 in cattle. Further investigations are needed to clarify the genetic characteristics of EHDV-6 HG-1/E/15 to determine why this outbreak occurred.
